# Lexical meaning is lower dimensional in psychosis

**DOI:** 10.1038/s41598-025-30443-1

**Published:** 2025-12-05

**Authors:** Claudio Palominos, Frederike Stein, Tilo Kircher, Rosa Ayesa-Arriola, Lena Palaniyappan, Philipp Homan, Iris E. Sommer, Wolfram Hinzen

**Affiliations:** 1 Department of Translation & Language Sciences, Universitat Pompeu Fabra, Barcelona, Spain; 2 Department of Psychiatry and Psychotherapy, University of Marburg, Marburg, Germany; 3 Center for Mind, Brain and Behavior (CMBB), Marburg, Germany; 4 Department of Psychiatry, Marqués de Valdecilla University Hospital, IDIVAL, School of Medicine, University of Cantabria, Santander, Spain; 5 Biomedical Research Center in Mental Health Network (CIBERSAM), Health Institute Carlos III, Madrid, Spain; 6 Department of Psychiatry, Douglas Mental Health University Institute, McGill University, Montreal, QC Canada; 7 Department of Medical Biophysics, Schulich School of Medicine and Dentistry, Western University, London, ON Canada; 8 Department of Psychiatry, Schulich School of Medicine and Dentistry, Western University, London, ON Canada; 9 Robarts Research Institute, Schulich School of Medicine and Dentistry, Western University, London, ON Canada; 10 Department of Adult Psychiatry and Psychotherapy, University of Zurich, Zurich, Switzerland; 11 Neuroscience Center Zurich, University of Zurich and ETH Zurich, Zurich, Switzerland; 12 Department of Neuroscience, University Medical Center Groningen, Antoni Deusinglaan 2, room 117, Groningen, Netherlands; 13 Institució Catalana de Recerca i Estudis Avançats (ICREA), Barcelona, Spain

**Keywords:** Schizophrenia, Psychosis, Language models, Semantics, Word embeddings, Mathematics and computing, Neuroscience, Psychology, Psychology

## Abstract

Diverse language models (LMs), including large language models (LLMs) based on deep neural networks, allow us to chart how people organize meanings in speech and how this process breaks down in conditions. Recent evidence has pointed to higher mean semantic similarities between words in people with psychosis, conceptualized as a ‘shrunk’ (more compressed) semantic space. Based on this, we hypothesized that the dimensionality of the vector spaces as defined by the embeddings of speech samples from LMs would also be easier to *reduce* in psychosis. To test this, we used principal component analysis (PCA) to calculate different metrics serving as proxies for reducibility, including the number of components needed to reach 90% of variance, and the cumulative variance explained by the first two components. For further exploration, intrinsic dimensionality (ID) was also estimated. Results consistent over datasets in three languages confirmed significantly higher reducibility of the semantic space in psychosis. This result points to the existence of an underlying intrinsic geometry of the space of semantic associations in speech, which may underlie more surface-level measurements such as semantic similarity. It also offers a new foundational approach to speech in mental disorders.

**Supplementary Information:**

The online version contains supplementary material available at 10.1038/s41598-025-30443-1.

## Introduction

When communicating, we draw concepts from our semantic memory into structured speech, using grammar to connect different words and phrases to convey meaning. Through this process, we metaphorically navigate a semantic space, traversing different regions in a trajectory that as such gives meaning to the discourse as a whole. This notion aligns with the framework of distributional semantics in language models (LMs), where (sub)words, sentences, or even larger linguistic units are represented as vector embeddings in a high-dimensional vector space. Metrics such as cosine similarity capture the relationships between vectors: the smaller the angle between them, the more semantically related the words, sentences, or texts are, as they tend to occur in similar contexts in the data used to train these models.

Several semantic similarity metrics have been employed to analyze spontaneous speech of people with schizophrenia spectrum disorder (SSD), who exhibit distinct navigational patterns through semantic space compared to healthy controls^1–5^. These differences have informed the development of classification models aimed at distinguishing clinical groups like people at high-risk who will/will not transition to psychosis and individuals with/without SSD^6,7^. At the core of this research program lies the question of whether major psychiatric disorders such as SSD disrupt the underlying organization of the semantic space and how this manifests in natural speech^8^.

Yet despite the growing use of semantic variables in recent studies, little is known about how these variables relate to each other, or whether they reflect overlapping or independent aspects of semantic organization. This lack of integration complicates efforts to interpret findings across studies or to identify robust, generalizable features of semantic disruption in psychosis. One attempt to address this was made in^9^, by capturing the contributions of over a hundred semantic variables derived from language models jointly, in a single ‘composite index’. Parallel to this aggregating approach, work is required that would target the internal organization of the semantic space, identifying specific variables that capture its disorganization in psychosis in a principled and mathematically quantifiable way.

This study aims to capture an aspect of the geometry of the high-dimensional space of embeddings derived from LMs and spontaneous speech in SSD. Specifically, we infer the geometry of a given speech sample using principal component analysis (PCA) and an estimation of intrinsic dimensionality (*ID*). While PCA finds an orthogonal basis in a given coordinate system that best explains variance in the data, *ID* identifies the minimum number of degrees of freedom needed to describe the data without significant loss of information^10^. Given that the lexical concepts of each speech sample refer to a specific picture description, a significant reduction in the *ID* with respect to the original word embeddings is expected. That is, the embeddings will have more information than what is needed for a given picture, and they share semantic properties that would allow to reduce this information into a lower dimensional space. In this context, *ID* provides an estimate of the number of latent degrees of freedom or abstract features required to encode the linguistic patterns within speech, offering insights into the structure of semantic expression.

Together, the number of principal components (PCs) required to explain a given amount of variance, the variance explained by the first components, and the values of *ID*, serve as mutually informative measures of the reducibility of the embeddings and how semantically associated the words are. When comparing two speech samples of equal length, a smaller number of components or a lower *ID* suggests that the shared information across the set of embeddings within each speech is more redundant, as it can be captured using fewer dimensions.

We here interpret this reducibility as a lexical phenomenon, arising from the relationships among content words that define the semantic space. Accordingly, we first used fastText embeddings, which represent words independently of context and thus capture lexical-conceptual meaning. In this framework, the geometry under study reflects the organization of lexical meaning. The secondary analyses using BERT embeddings, which include both content and functional words, extend this perspective by situating lexical items within grammatical and contextual constraints.

While the focus in our analysis is primary lexical, ultimately lexical selection is entangled in speech with grammatical structure. Content words do not occur in isolation but are embedded in syntax, meaning that the observable lexical patterns also reflect grammatical organization to some extent. This interdependence motivates our further exploration of *ID* across BERT layers, a contextual large language model, where early layers capture more surface features, middle layers syntactic information and later ones encode more abstract and semantic relationships^11^. Examining how *ID* varies across layers provides a complementary, multi-scale view of how lexical and grammatical constraints shape the latent structure of language in SSD.

We hypothesized that the intrinsic geometry of the semantic space in SSD is lower-dimensional compared to that of HC, which aligns and complements the observed patterns of a ‘shrunk’ semantic space as manifest through increased mean semantic similarity^3,12,13^, or a reduced convex hull volume (i.e., the volume of the convex shape enclosing the core of the data) in SSD^3^. In line with this hypothesis, we found across three datasets and three languages, and controlling by demographics including sex, age and education, and number of words, that SSD speech is more reducible compared to healthy controls, whether using PCA or *ID*. This result illustrates a new principled approach, focussed on the geometry of the semantic space through observable word embeddings, which may help to explain the previously observed pattern of a more compressed semantic space, becoming more semantically interconnected in SSD.

## Methods

### Data collection

The datasets comprised speech samples from German (*N* = 86), Spanish (*N* = 84) and English (*N* = 94) speakers. Demographics and clinical data are summarized in Table 1, including the different compositions of subsamples. In the English sample, SSD was divided into first-episode psychosis (FEP) and chronic schizophrenia (CS) to examine potential differences within SSD, as well as a clinical high-risk (CHR) group. In each language, the samples were obtained through the same task of describing pictures from the Thematic Apperception Task (TAT). This test provided the opportunity to control that participants describe specific topics, limiting thematic variability. Full details of these German, Spanish, and English samples can be found in^4,14^, and^3^, respectively. For all the samples, data collection, protocol and methodology were approved by the local ethical committees (German: Ethik-Kommission des Fachbereichs Humanmedizin der Philipps-Universität Marburg (AZ 07:14); Spanish: Comité de Ética de la Investigación con Medicamentos (CEIm) de Cantabria (2021.119); English: The Research Ethics Committee of University of Western Ontario, London, Canada (Project ID: 108268; Most recent review reference: 2022–108268-71496; Study Title: The Pathophysiology of Thought Disorder in Psychosis (TOPSY)). An informed consent was obtained from all subjects and/or their legal guardian(s). All methods were performed in accordance with the relevant guidelines and corresponding regulations.

**Table 1:** Demographics of participants and comparisons for each sample.

German	HC (mean ± SD)	SSD (mean ± SD)	HC – SSD (*p*-value)	
Nº participants	44	42		
Age (years)	42.9 ± 12.3	40.5 ± 11.9	0.3766	
Female (%)	32%	38%	0.7008	
Education (years)	14.7 ± 3.3	11.9 ± 2.0	0.0000***	
IQ	117.5 ± 14.2	111.6 ± 15.9	0.0786	
Age of onset (years)	-	17.7 ± 8.2	-	

**Spanish**	**HC** **(mean ± SD)**	**SSD** **(mean ± SD)**	**HC - SSD** **(p-value)**	
Nº participants	41	43		
Age (years)	41.3 ± 7.8	38.1 ± 9.0	0.0844	
Female (%)	41.5%	48.8%	0.6459	
Education (years)	11.6 ± 2.6	10.5 ± 2.0	0.1145	

**English**	**HC** **(mean ± SD)**	**CS** **(mean ± SD)**	**CHR** **(mean ± SD)**	**FEP** **(mean ± SD)**	
Nº participants	29	18	18	29	
Age (years)	21.5 ± 3.6	28.5 ± 7.4	22.4 ± 4.1	21.4 ± 2.2	
Female (%)	24.1%	22.2%	27.7%	27.6%	

	**HC – CS** **(p-value)**	**HC – CHR** **(p-value)**	**HC – FEP** **(p-value)**		
Age (years)	0.0000***	0.4464	0.8606		

	**CS – CHR** **(p-value)**	**CS – FEP** **(p-value)**	**CHR – FEP** **(p-value)**		
Age (years)	0.0047	0.0000***	0.2817		

Note: IQ scores are derived from the MWT-B scale, which assesses verbal premorbid intelligence. Gender comparisons were performed using the χ² test, while t-tests were used for comparing other variables. Significance levels are denoted as follows: * *p* <.05, ** *p* <.01, *** *p* <.0001.

### Word embeddings

300-dimensional word embeddings for each speech sample were obtained using fastText^15^, and 768-dimensional embeddings were obtained using BERT (Bidirectional Encoder Representations from Transformers^16^;. When fastText was used, unique content words were retained to avoid measuring effects related to lexical diversity, while stopwords and punctuation were removed. However, in a post-hoc analysis, all content words were retained. Preprocessing was carried out using spaCy (version 3.6.0), and the models ‘de_core_news_lg’ for German, ‘es_core_news_lg’ for Spanish, and ‘en_core_web_lg’ for English were applied. When BERT was used, all words were retained, and the models ‘dbmdz/bert-base-german-cased’^17^ for German, ‘dccuchile/bert-base-spanish-wwm-cased’^18^ for Spanish, and ‘bert-base-cased’^16^ for English were employed.

### Principal components analysis

A principal component analysis (PCA) was conducted on the set of embeddings for each speech sample, with columns as the number of dimensions and rows for the number of words in the sample. This allowed each speech sample to be represented by a matrix with fewer dimensions, using the principal components of the word embeddings, instead of the original 300 (or 768 for BERT), while retaining most of the variance. From the PCA, we derived variables indicating the amount of variance explained by the principal components. To do this, we defined $\:{Ncomp}_{x}$ as the number of components required to explain $\:x$ percent of variance, and $\:{ExVar}_{n}$ the cumulative explained variance by the first $\:n$ principal components. For group comparisons, we specifically used $\:{Ncomp}_{90}$ and $\:{ExVar}_{2}$, capturing both ends of the components. However, both functions ($\:{Ncomp}_{x}$ and $\:{ExVar}_{n}$) proved effective in signalling group differences across different levels of $\:x$.

### Intrinsic dimensionality

Intrinsic dimensionality (*ID*) is the lowest number of dimensions required to represent a given dataset without significant information loss. It captures the underlying structure of high-dimensional data and serves as a proxy for reducibility^10^. Since linear techniques such as PCA may not effectively capture nonlinear relationships within the data, a variety of alternative dimensionality reduction methods have been proposed^19^.

In this study, we estimated the intrinsic dimensionality of the embeddings for each speech sample using the Maximum Likelihood Estimation (MLE) method implemented in *scikit-dimension*^20^. This approach models the distances to nearest neighbors for each point in the dataset, assuming that the data lie on a single manifold^21^. For each point, a local maximum likelihood estimate of dimension is calculated, and these local estimates are then aggregated to obtain a global intrinsic dimension. The procedure involved first obtaining the word embeddings for each speech sample and then applying MLE to estimate the ID from their correlation structure. This yielded a single *ID* value for each speech sample. Additionally, we conducted a sensitivity analysis estimating *ID* with different values of $\:k$ in the $\:k$-nearest neighbors procedure (Figures S4 and S5 in SM).

### Statistical analysis

To determine group differences between reducibility variables, we used different models based on the sample, controlling for number of words, education (except the English sample, where education was a categorical variable), age, and gender in all cases. We used a mixed-effects model to estimate population-averaged effects while controlling for picture effects, which have previously been shown to be significant^4^. When the assumptions of normality were violated, we instead applied fixed-effects-only models. In such cases, values were averaged across samples (Spanish and English). Specifically, for each variable ($\:{Ncomp}_{90}$, $\:{ExVar}_{2}$ and *ID*), we estimated the coefficients of the independent variables SSD, CS, FEP, and CHR (dummy variables indicating group membership), as well as number of content words, number of tokens, gender (female), age, and education (see Eqs. (1), (2), and (3), for German, Spanish and English samples, respectively). Here, $\:{y}_{ij}$ denotes the dependent variable ($\:{Ncomp}_{90}$, $\:{ExVar}_{2}$ or *ID*), with $\:i$ indexing participants and $\:j$ indexing different observations within participants. The error structure includes a subject-specific random intercept $\:{\mu\:}_{i}$ to account or within-participant dependence, and a residual error term $\:{\varepsilon\:}_{ij}$. In the BERT analysis, the number of content words variable was replaced by the number of tokens. To rule out the effect of differing word counts, we not only controlled for this in the models but also ran a t-test to verify that there were no significant differences between groups within each sample (see SM, Figure S1).

Finally, we did not apply multiple-comparison corrections across regressions because the models tested related outcomes (*ID*, $\:{Ncomp}_{90}$, $\:{ExVar}_{2}$), which represent complementary indices of the same underlying construct. These measures are theoretically and empirically correlated, and applying corrections that assume independence would therefore be overly conservative and risk inflating Type II errors. Instead, our analyses focus on convergent patterns replicated across independent datasets, languages, and measures, providing a more robust assessment of the underlying phenomenon than isolated significance values. All the calculations and coding were performed in Python (version 3.12.4).

1 $$\begin{aligned}\:{y}_{ij} & ={\beta}_{0}+{\beta}_{SSD}\times\:{SSD}_{ij}+{\beta}_{2}\times\:{picture}_{2}+{\beta}_{4}\times\:{picture}_{4}+{\beta}_{6}\times\:{picture}_{6}+{\beta}_{words}\\ & \times\:{n\_words}_{ij}+{\beta}_{age}\times\:{age}_{ij}+{\beta}_{female}\times\:{female}_{ij}+{\beta}_{education}\\ & \times\:{education}_{ij}{\:+\:{\mu\:}_{i}\:+\:\varepsilon}_{ij}\end{aligned}$$

2 $$\begin{aligned}\:{y}_{i}&={\beta}_{0}+{\beta}_{SSD}\times\:{SSD}_{i}+{\beta}_{words}\times\:{n\_words}_{i}+{\beta}_{age}\times\:{age}_{i}+{\beta}_{female}\\ & \times\:{female}_{i}+{\beta}_{education}\times\:{education}_{ij}{\:+\:\:\varepsilon}_{i}\end{aligned}$$

3 $$\begin{aligned}\:{y}_{i}&={\beta\:}_{0}+{\beta}_{CS}\times\:{CS}_{i}+{\beta}_{FEP}\times\:{FEP}_{i}+{\beta}_{CHR}\times\:{CHR}_{i}+{\beta}_{words}\times\:{n\_words}_{i}+{\beta}_{age}\\ & \times\:{age}_{i}+{\beta}_{female}\times\:{female}_{i}{+\:\:\varepsilon}_{i}\end{aligned}$$

### Correlation between measures

We calculated the pairwise Pearson correlation between all measures and the average cosine similarity between consecutive words (hereafter, *semantic similarity*), and the dispersion with respect to the centroid of word embeddings (hereafter, *dispersion*), both using *fastText*.

## Results

A preliminary analysis showed that $\:{Ncomp}_{90}$ was strongly correlated with the number of content words (Pearson correlation, *r* =.905). This strong correlation is anticipated, given that word embeddings are already low-rank representations. However, we confirmed this expected relationship to ensure robustness in subsequent analyses. An equivalent pattern was found between $\:{ExVar}_{2}$ and the number of content words (Pearson correlation, *r* = -.519) which means that with more words less variance is explained by the first components. Both metrics, including related ones (such as various thresholds of variance explained, or the variance accounted for by a certain number of components), indicate an expected decrease in reducibility as the number of content words increases. Although no significant differences were found in the number of words when comparing groups in each sample (Figure S1), the correlation between word count and our metrics supports its inclusion as a covariate in the regression models, and our use of time-limited picture description task instead of open interviews, providing a finite constraint for content words.

### Regression models in different samples

#### Non-contextual model: fasttext

**Fig. 1:**
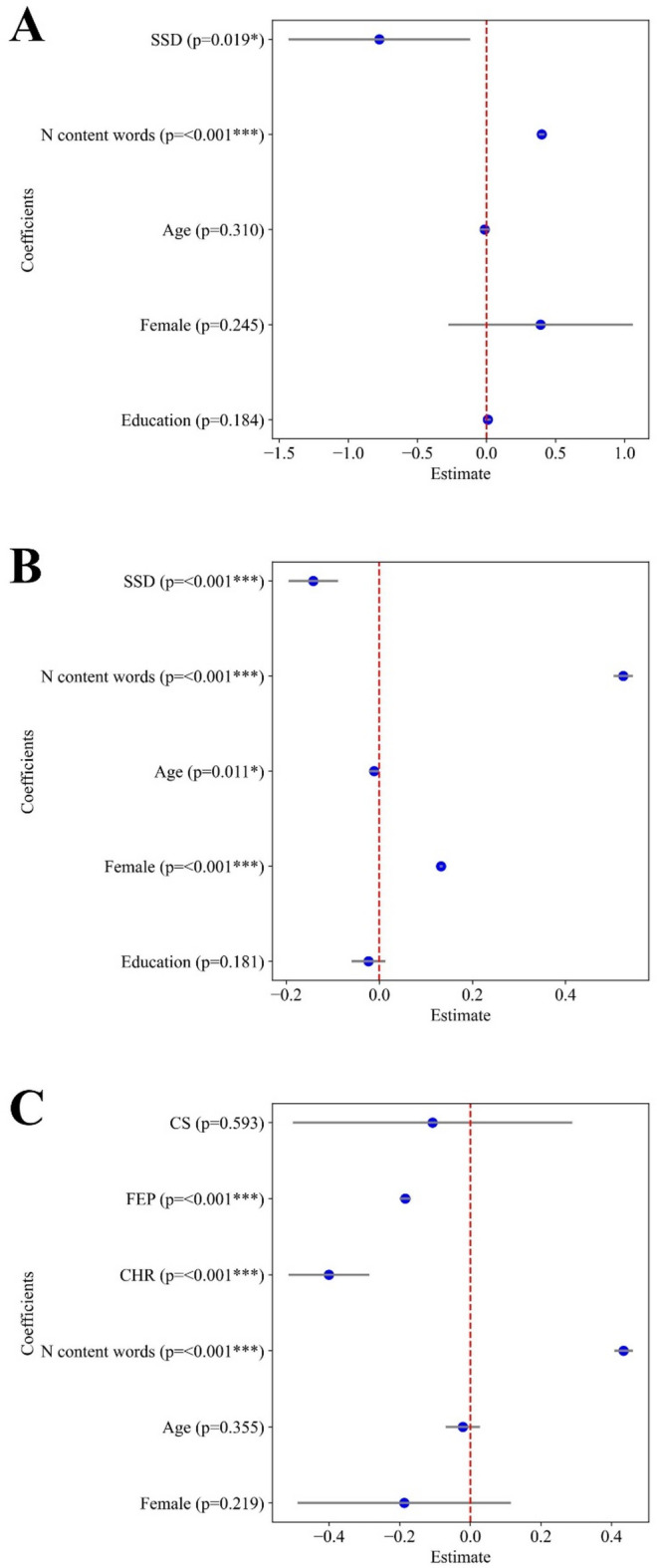
Estimated regressions coefficients for $\:{Ncomp}_{90}$ across languages. Panel **A**: German sample; Panel **B**: Spanish sample; Panel **C**: English sample. Each blue dot indicates the estimated coefficient, with horizontal lines representing the 95% confidence interval (0.025–0.975). P-values are shown next to each regressor. SSD: Schizophrenia Spectrum Disorder. CS: Chronic schizophrenia. CHR: Clinical High Risk FEP: First episode psychosis (untreated).

**Fig. 2:**
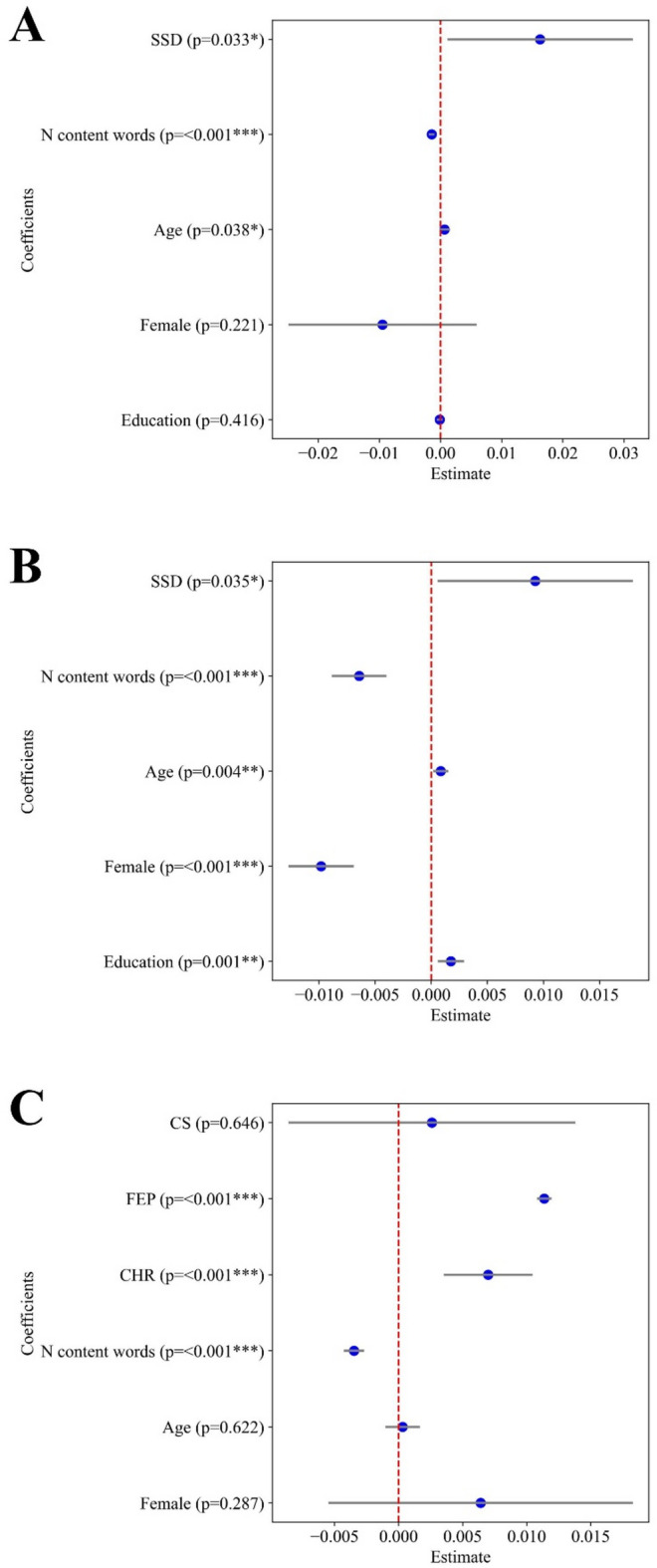
Estimated regressions coefficients for $\:{ExVar}_{2}$ across languages. Panel **A**: German sample; Panel **B**: Spanish sample; Panel **C**: English sample. Each blue dot indicates the estimated coefficient, with horizontal lines representing the 95% confidence interval (0.025–0.975). P-values are shown next to each regressor. SSD: Schizophrenia Spectrum Disorder. CS: Chronic schizophrenia. CHR: Clinical High Risk. FEP: First episode psychosis (untreated).

Figure 1 presents the model results for fastText model, showing that when controlling by picture, SSD and FEP samples required smaller $\:{Ncomp}_{90}$. Figure 2 presents equivalent results for the regression of $\:{ExVar}_{2}$, which shows that the first two components explain more variance in SSD and FEP compared to HC. Taken together, these results suggest that the word embeddings derived from speech in the SSD group are more reducible than those in the HC group. Fewer principal components were required to represent the original 300-dimensional embeddings in SSD compared to HC. An equivalent result is presented in Figure S2 and Figure S3, where all words are preserved rather than only the unique words.

#### Contextual model: BERT

**Fig. 3:**
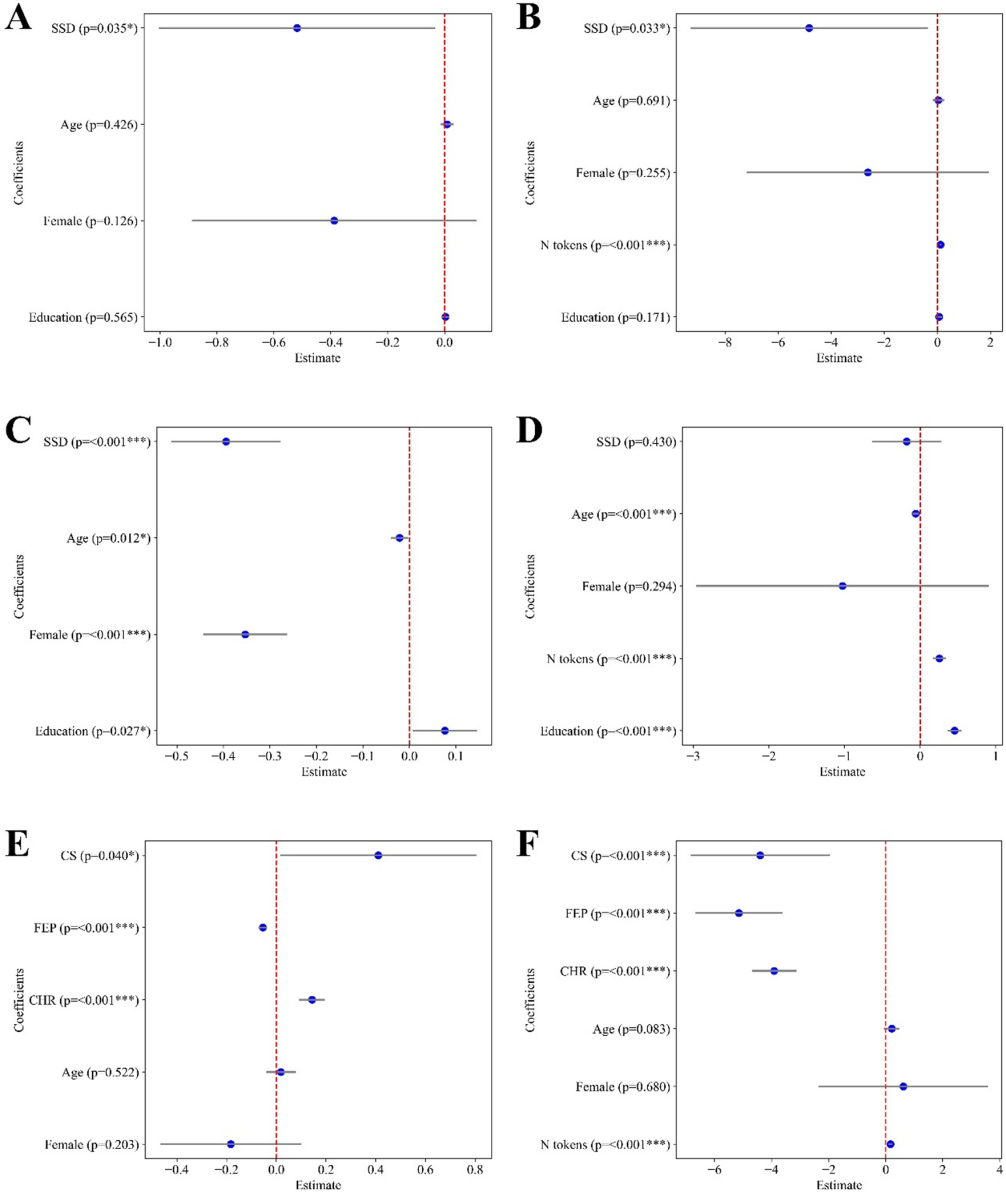
Estimated regression coefficients for *ID* and $\:{Ncomp}_{90}$ across languages using BERT embeddings. Panels **A** and **B**: German sample; Panels **C** and **D**: Spanish sample; Panels **E** and **F**: English sample. Left panels (**A**,** C**, and **E**) show estimated coefficients from models regressing *ID*, while right panels (**B**,** D**,** F**) show the estimated coefficients from models regressing $\:{Ncomp}_{90}$. Each blue dot indicates the estimated coefficient, with horizontal lines representing the 95% confidence interval (0.025–0.975). P-values are shown next to each regressor. SSD: Schizophrenia Spectrum Disorder. CS: Chronic schizophrenia. CHR: Clinical High Risk. FEP: First episode psychosis (untreated).

Figure 3 shows the model results for *ID* (Panels A, C, E) and $\:{Ncomp}_{90}$ (Panels B, D, F), both using BERT embeddings. *ID* was significantly lower in individuals with SSD compared to HC in both the German and Spanish samples. In the English sample, *ID* was significantly lower in individuals with FEP compared to HC. However, in the same sample, *ID* was significantly higher in individuals with CS and CHR compared to HC. This discrepancy suggests a divergence between results obtained using PCA-based measures and those based on *ID* in the English sample. The sensitivity analysis confirmed convergence of results after estimating *ID*, showing that larger $\:k$ values yield stronger significance (see SM, Figures S4, S5).

To further address these differences, $\:{Ncomp}_{90}$ was found to be significantly lower in all clinical groups compared to HC, except in the Spanish sample, where no significant differences were observed.

### Intrinsic dimensionality across BERT layers

**Fig. 4:**
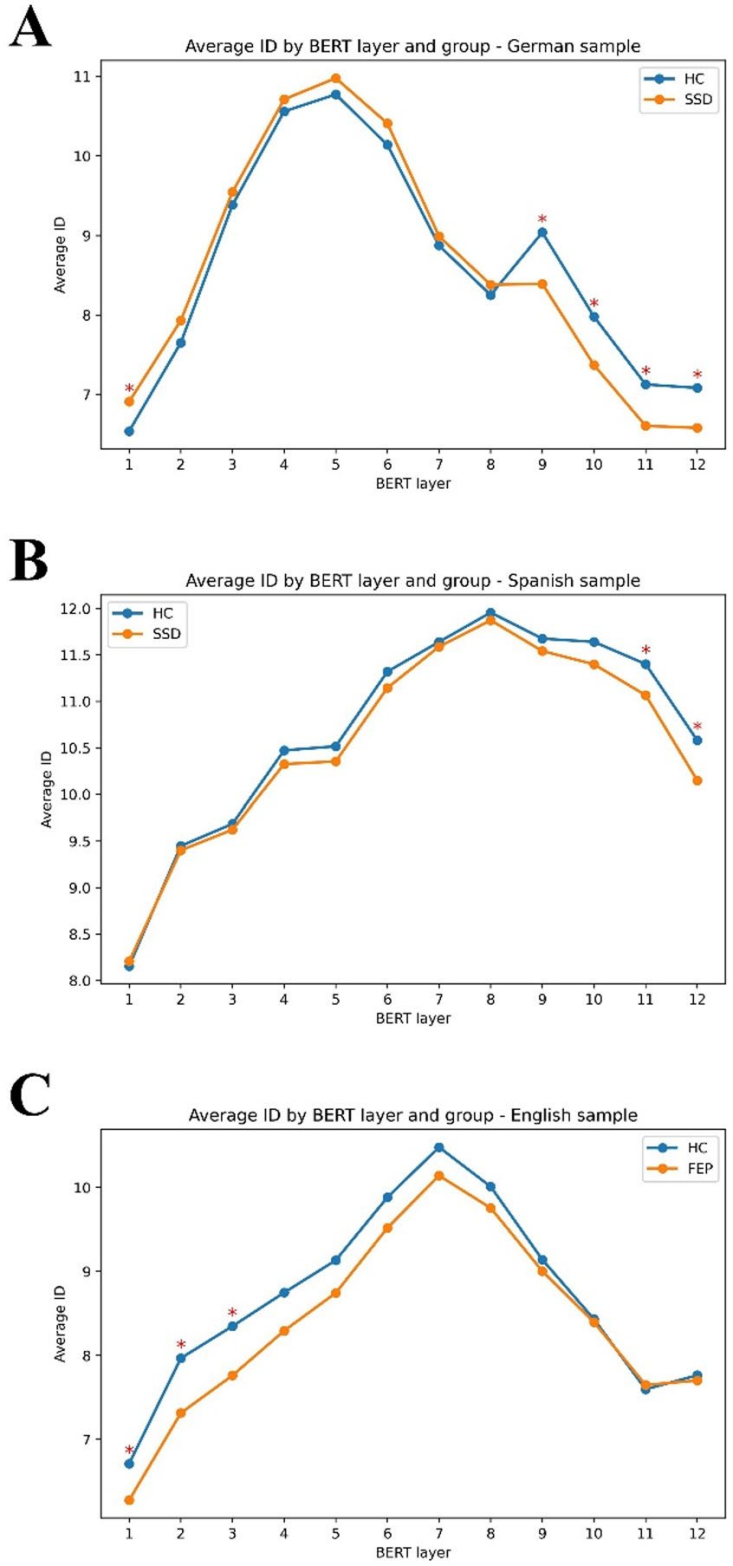
Average Intrinsic Dimensionality (*ID*) estimates across BERT layers for each group and sample. *ID* was computed layer-by-layer using 12-layer BERT embeddings for each group. Panel **A**: German sample; Panel **B**: Spanish sample; Panel **C**: English sample. Red asterisks indicate t-test statistically significant group differences at specific layers. Note that in the Spanish sample, only FEP and HC groups are compared here.

Figure 4: shows the average *ID* estimates for each group across the three languages using BERT-base (12 layers). Notably, only in the German sample significant groups differences appear after layer 6. Overall, *ID* is significantly lower in SSD groups compared to HC in both German and Spanish samples, and significantly lower in FEP and CHR groups, compared to HC in the English sample.

### Correlation between measures

**Fig. 5:**
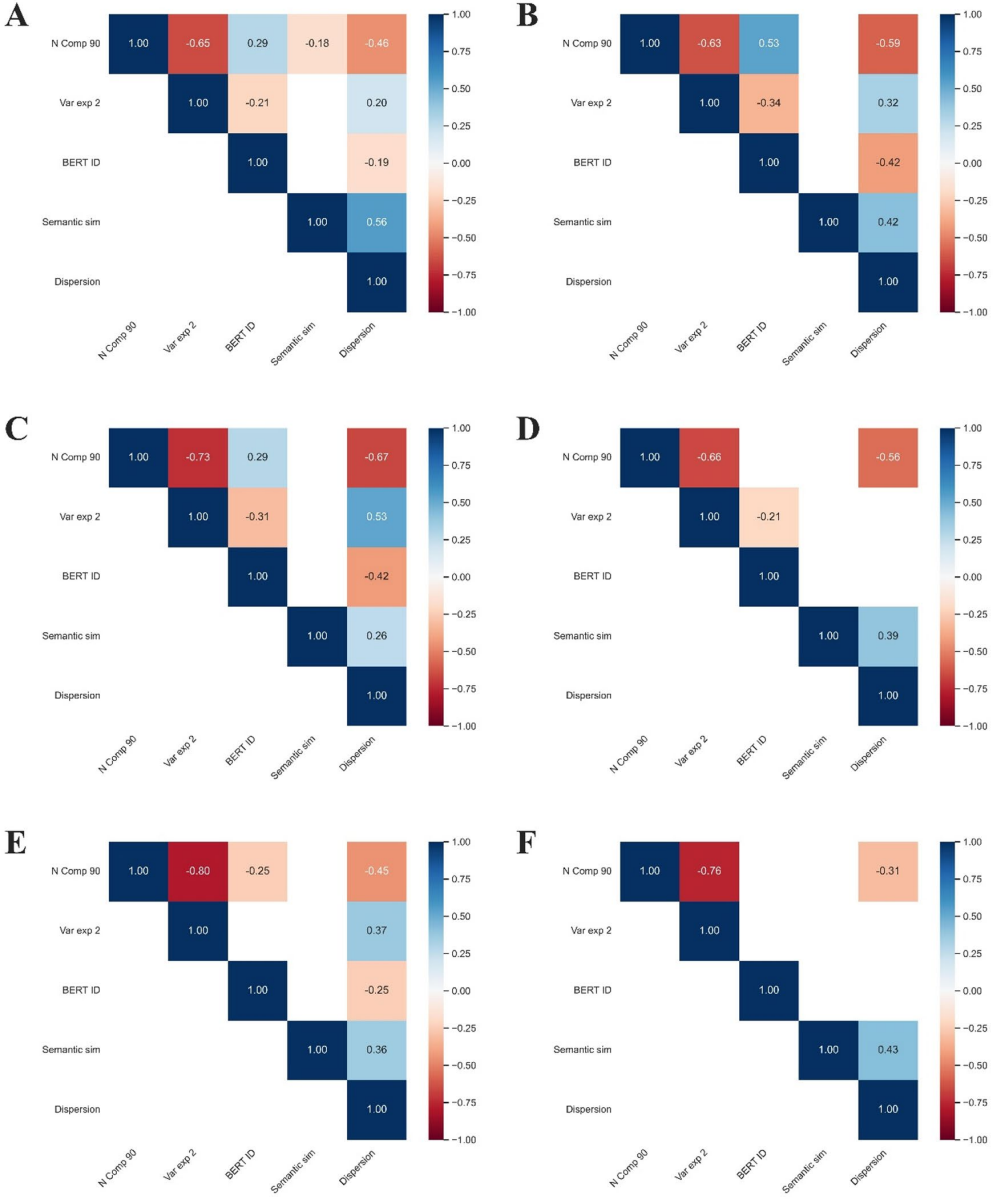
Correlation matrices of variables by language and group. Each heatmap shows pairwise Pearson correlation between variables for German (**A-B**), Spanish (**C-D**), and English (**E-F**) samples. Left panels (**A**,** C**, and **E**) show results for clinical groups (SSD or FEP), while right panels (**B**,** D**, and **F**) show HC. Only statistically significant correlations are shown (*p* <.05), with red indicating negative and blue indicating positive correlations. **N Comp 90**: Number of principal components required to explain 90% of the variance. **Var exp 2**: Variance explained by the first two principal components. **BERT** ***ID***: Intrinsic dimensionality estimated from BERT embeddings. **Semantic sim**: Mean cosine similarity between consecutive content words in a speech sample, *fastText*. **Dispersion**: Dispersion of all word embeddings with respect to its centroid, using *fastText*.

We calculated Pearson correlations coefficients between five variables: mean semantic similarity, dispersion to the centroid, $\:{Ncomp}_{90}$, $\:{ExVar}_{2}$, and *ID*. The resulting correlation patterns for each group are shown in Fig. 5. As expected, $\:{Ncomp}_{90}$ and $\:{ExVar}_{2}$ are strongly negatively correlated: if fewer components are needed to explain 90% of variance, it is likely that the variance explained by the first two components is higher. This correlation is weaker in the German sample, suggesting that the two measures may be capturing different aspects of reducibility in different samples. $\:{Ncomp}_{90}$ and *ID* were positively correlated only in the German sample, which support the idea that both measures capture the geometry of the semantic space in different ways. A similar pattern was found between $\:{ExVar}_{2}$ and *ID*, were both the German and Spanish samples showed a weak negative correlation. Additionally, only in the SSD group of the German sample did $\:{Ncomp}_{90}$ correlate negatively, and weakly, with mean semantic similarity.

## Discussion

Recent studies have shown a pattern of increase in semantic similarity measures in SSD compared to HC^1,3,12,13^, conceptualized as a ‘shrunk’ (more compressed) semantic space in SSD. Using word embeddings from both static (fastText) and contextual (BERT) models across three distinct languages (German, Spanish, and English datasets), we found that the dimensions of embeddings in SSD speech samples were significantly more reducible than those of HC, as measured by a set of variables including the number of principal components required to explain variance, the variance explained by early components, and intrinsic dimensionality (*ID*). Importantly, these measures in part reflect the narrower lexical field inherent to the picture description task. However, beyond task-related lexical constraints, the additional reduction observed in clinical groups, and converging findings across languages support the interpretation that SSD speech exhibits more semantic redundancy and reduced semantic variability, as systematically fewer dimensions are needed to represent the words used.

Although our primary aim was to investigate reducibility in lexical meaning using a non-contextual model like fastText, we extended this approach to a contextual model (BERT), which captures all words, including functional ones, and encodes richer semantic nuances through higher-dimensional representations. The results generalized across models and languages in symptomatic patients (SSD and FEP). This effect may be sensitive to the stage of psychosis as CS and CHR subjects showed higher *ID* than HC in the English sample. Stage specific differences in language profile have already been shown in^22^.

Additionally, in the Spanish sample, $\:{Ncomp}_{90}$ in BERT analysis, did not differ significantly from HC. One possible explanation is that lexical reducibility using fastText may underlie the reducibility of the semantic space in SSD, whereas this effect may be diluted in BERT embeddings, which integrate broader contextual information and non-lexical elements. Further research is needed to address this and disentangle the relation between effects of different models and reducibility measures.

It is worth noting that the English sample presents specific characteristics that may account for some of the observed variability. The FEP group, which showed the expected reduction in *ID*, is the largest (*n* = 29), most symptomatic, largely unmedicated, and severe one of the clinical groups. In contrast, the chronic group (*n* = 18) was remitted and exhibited remarkably low symptoms, whereas individuals at clinical risk (*n* = 18) tended to be a highly heterogeneous group.

Some apparently divergent findings in the English sample (Fig. 3, Panels E and F) suggest that the BERT *ID* in CS may be higher than in HC (Fig. 3, Panel E). We interpret this as a consequence of including more words and their contexts, which introduces additional contextual variability. By contrast, the $\:{Ncomp}_{90}$ results with BERT (Fig. 3, Panel F), significantly lower for all clinical groups compared to HC, appear consistent with the FastText *ID* results, as the variance introduced by contextual information is largely filtered out by the principal components explaining 90% of the variance. Overall, we believe that the semantic space derived from content words alone reveals that the reducibility is primarily lexical, and that the BERT results may reflect this as well.

To gain more insight into the architecture of BERT’s hidden layers, we estimated *ID* on a layer-by-layer basis. All the samples and groups showed a similar pattern of increase of *ID* in the early layers, with a peak at a certain layer, and declining thereafter, although differing in the location of the peak. For German and Spanish samples, differences between groups appeared in later layers where semantic relationships are supposed to be encoded, in contrast to the earlier layers that encode more surface-level linguistic features^11^. This suggests that the reducibility of the embeddings space defined by words in speech occurs primarily at a semantic level, although it may also be modulated by grammatical structure.

The notion of semantic reducibility must be distinguished from semantic similarity as previously studied in the literature. While these two phenomena may be related, our correlation analyses revealed that they are not trivially equivalent. The mixed patterns found in the correlation analysis suggest that semantic reducibility captures more than just surface-level similarity between words, and it may reflect the intrinsic geometry of the semantic space of speech, as well as the grammatical constraints that are entangled with specific lexical selections in a particular speech sample. Additionally, to further explore the relationship between semantic similarity and reducibility measures, it would be necessary to compare individuals with the same speech lengths and assess whether these associations vary by clinical group.

It is worth considering that the reducibility of the semantic space may partly reflect underlying grammatical constraints. While our analyses focused on content words, lexical selection in speech is tightly coupled with grammar. In this context, the observed geometric properties may not only stem from lexical choices but also from the grammatical scaffolding that shapes them. A possible direction for future research would be to examine the contribution of grammatical features to the reducibility of the semantic space across clinical groups.

From a broader perspective, this approach offers both a methodological and conceptual advance. Rather than quantifying differences derived from the embeddings themselves, it models the geometry of the representational space itself. By focusing on the number of dimensions required to represent words, we target a more fundamental property of language organization. We argue that this reducibility, which reflects how lexical variability is structured within the semantic space, provides a stronger and more integrated signal of semantic organization than measures such as similarity alone. Moreover, these findings underscore the need for caution when applying dimensionality reduction as a preliminary step to compute other measures, since speech samples with different intrinsic dimensionalities may be unevenly distorted in a common reduced space, thereby introducing noise and bias in downstream analyses.

Further, clinical status may influence semantic reducibility. For example, in the English sample, the FEP group showed more reducibility compared to HC, while CS patients, who presented with relatively mild symptomatology at the time of recording (see^3^, did not differ significantly from HC. This finding aligns with the idea that reducibility effects are most pronounced during early (and in this case acute) phases of psychosis, and supports a phase-dependent interpretation, where alterations in the semantic structure of speech are more clearly observed during acute illness stages and may diminish with clinical stability or remission^22^. Lexical reducibility in this context may reflect cognitive rigidity and associative constraint, particularly pronounced in FEP.

At the same time, it is important to acknowledge that the semantic space derived from language embeddings represents a limited projection of meaning. In reality, meaning arises from a mixture of qualitatively distinct representational systems whose interactions cannot be fully captured by the vector geometries used here. This limitation is not merely conceptual but methodological, as it constrains which aspects of meaning distortion in psychosis can be captured with current models. Future work should therefore aim to integrate complementary representational frameworks capable of reconcile the interplay between different components of meaning, such as, for instance, the interaction between conceptual-semantic and referential organization in speech.

Finally, while our study focused on BERT-base and *fastText*, future research should explore whether the same effects hold when using larger and more diverse models, such as LLaMA, Mistral, or Gemma. Expanding this research across more languages and embedding architectures would provide further insights into the cross-linguistic generalizability and computational robustness of the reducibility phenomenon.

## Conclusions

Exploring the semantic space in psychosis has led to a number of computational measures showing sensitivity in different samples and languages, yet results consistent over several languages have proved difficult to achieve. The present study has explored dimensionality reduction as a new metric, which extends previous measures related to semantic similarity, in a geometric approach to the semantic space. This approach has revealed a pattern of reduced dimensionality in speech in psychosis, generalizable across SSD samples in three languages, different model architectures, and metrics exploring the same basic concept. This provides a proof of this concept, which now requires further validation in samples reflecting different disease stages, using different model architectures.

## Supplementary Information

Below is the link to the electronic supplementary material.

Supplementary Material 1

## Data Availability

Anonymised transcripts of TOPSY study data are made available to qualified researchers through https://talkbank.org/psychosis/, a collaboration between DISCOURSE in Psychosis consortium (https://discourseinpsychosis.org/) and TalkBank. Restrictions apply and conditions are accessible via the TalkBank URL above. Other data are available upon reasonable request contacting the corresponding author.
