# Supplementary material for: Lexical meaning is lower dimensional in psychosis
Source: Sci Rep. 2025 Dec 5;16:859. doi: 10.1038/s41598-025-30443-1 (PMC12780149; doi:10.1038/s41598-025-30443-1)
Supplement: Supplementary file 1 — Supplementary Material 1 [file 41598_2025_30443_MOESM1_ESM.docx]

**Supplementary Materials**

**Number of words: comparison between groups**

To rule out potential effects of differing word counts in the regression analyses, we ran *t*-tests to assess group differences within each sample. Figure S1 shows boxplots of word count across groups and samples. No significant differences were observed.

| A  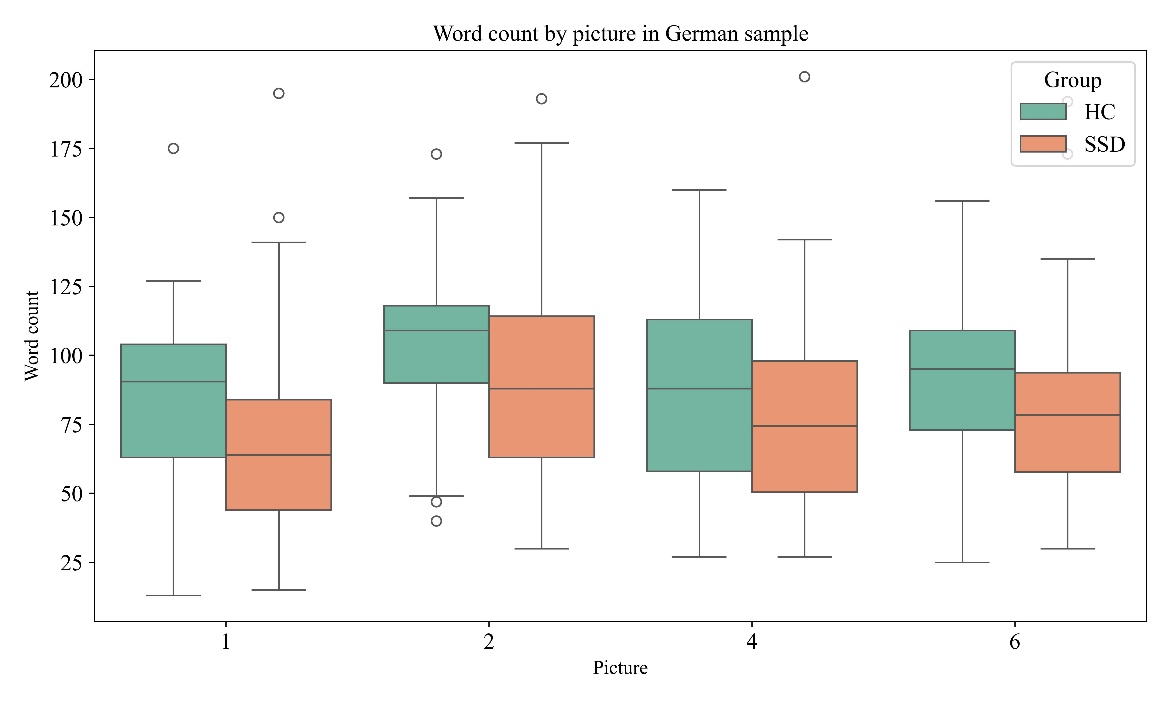 |
| --- |
| B  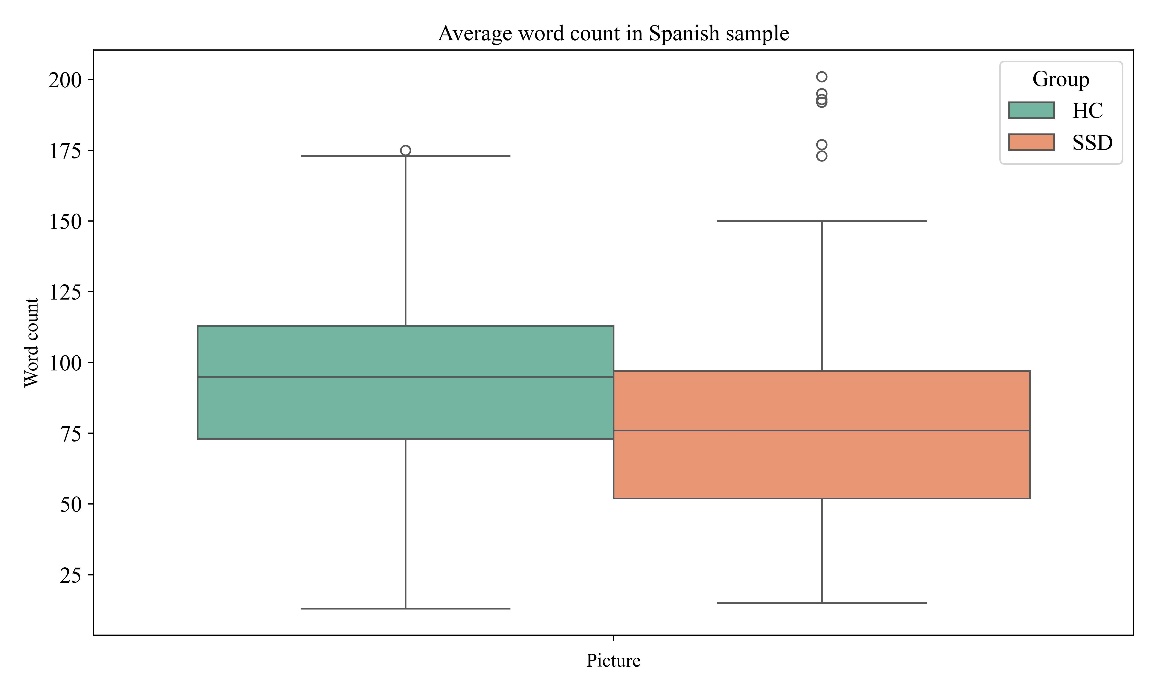 |
| C  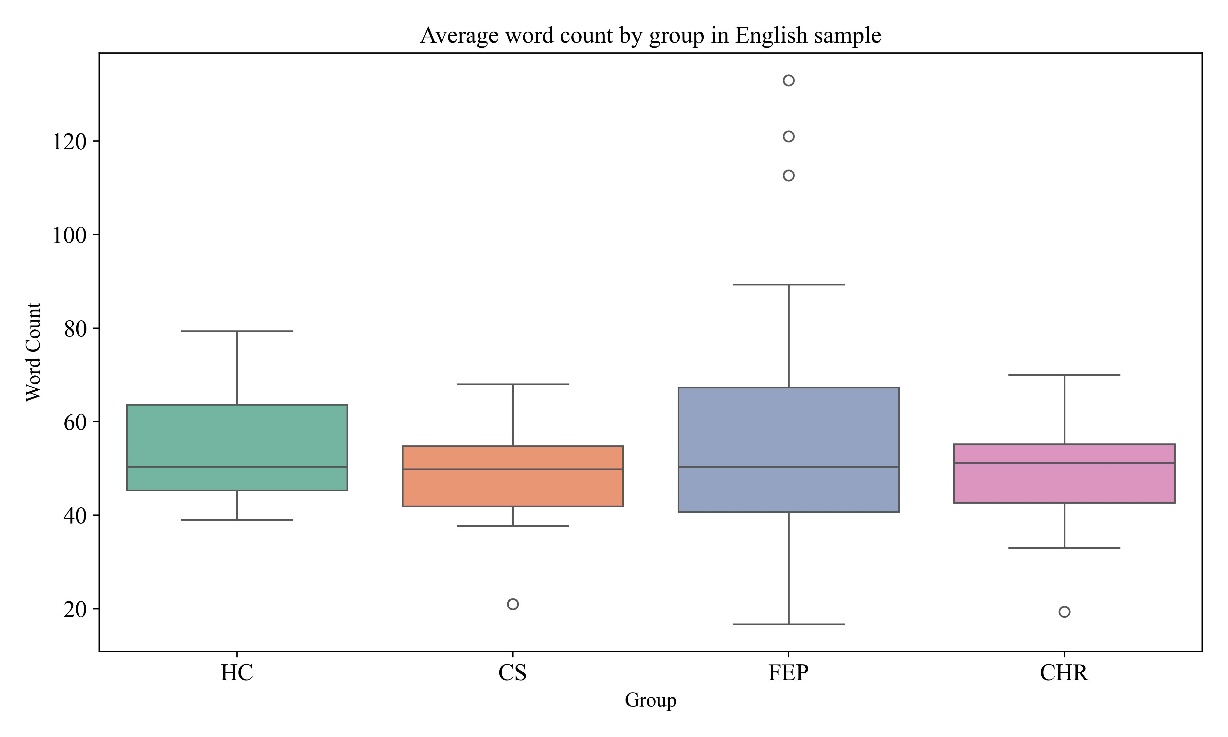 |

**Figure S1**: Boxplot of number of words across samples and groups. (A) German, (B) Spanish, (C) English. No significant differences in number of words were found when comparing groups. SSD: Schizophrenia Spectrum Disorder. CS: Chronic schizophrenia. CHR: Clinical High Risk. FEP: First episode psychosis (untreated)

**Regression models: additional results**

| A  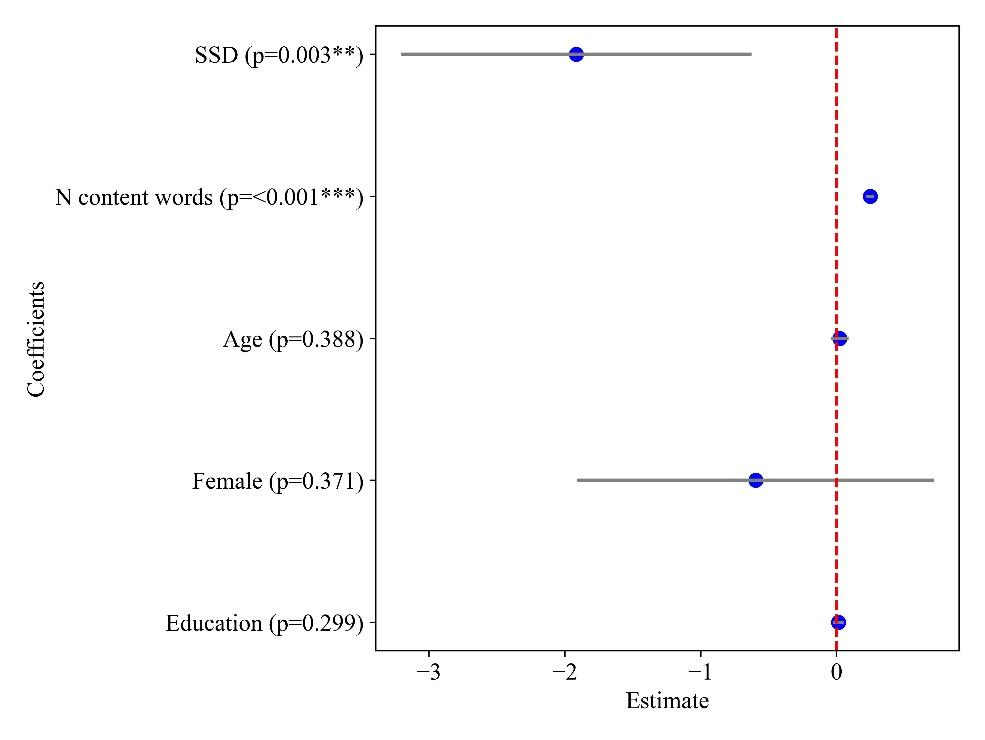 |
| --- |
| B  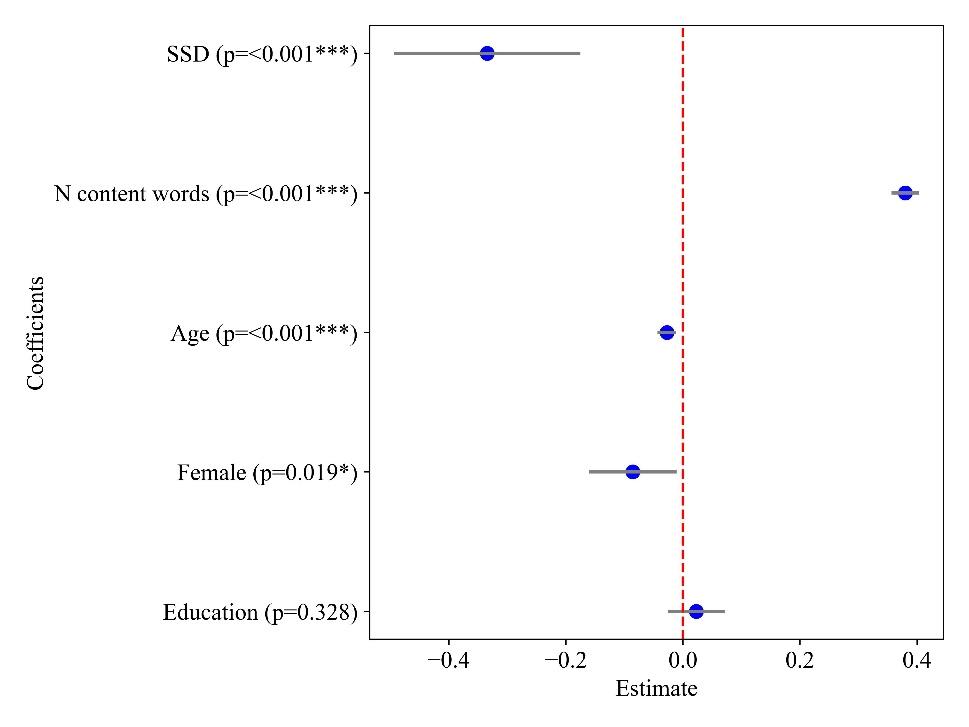 |
| C  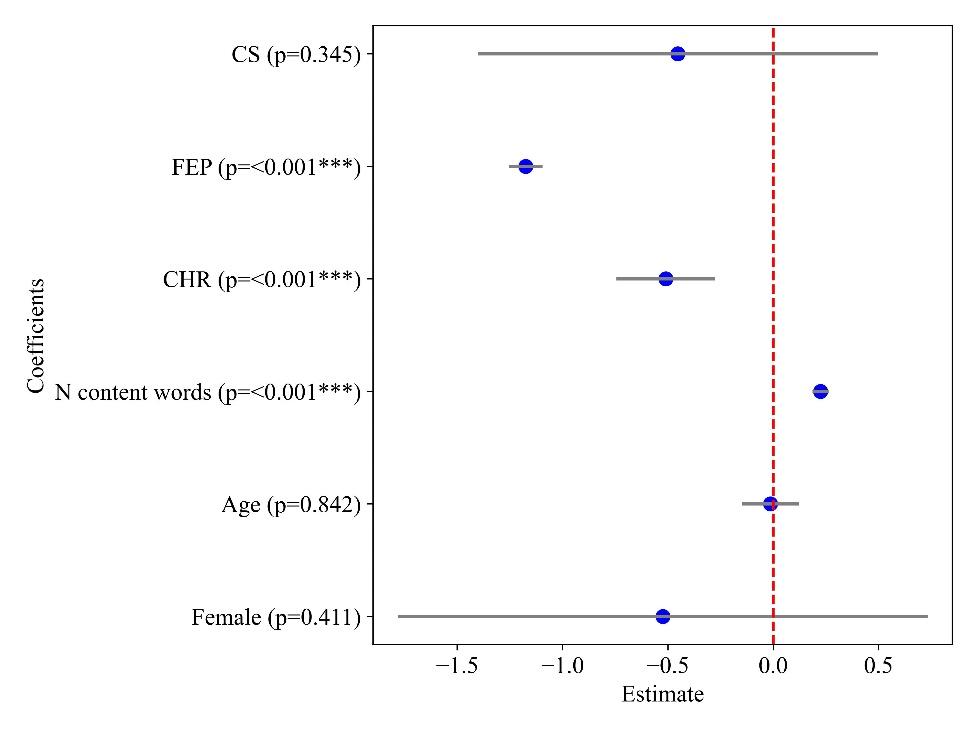 |

**Figure S2**: Estimates of coefficients for each language and regression model of ${Ncomp}_{90}$ using all content words in fastText. (A) German, (B) Spanish, (C) English. SSD: Schizophrenia Spectrum Disorder. CS: Chronic schizophrenia. CHR: Clinical High Risk. FEP: First episode psychosis (untreated).

| A  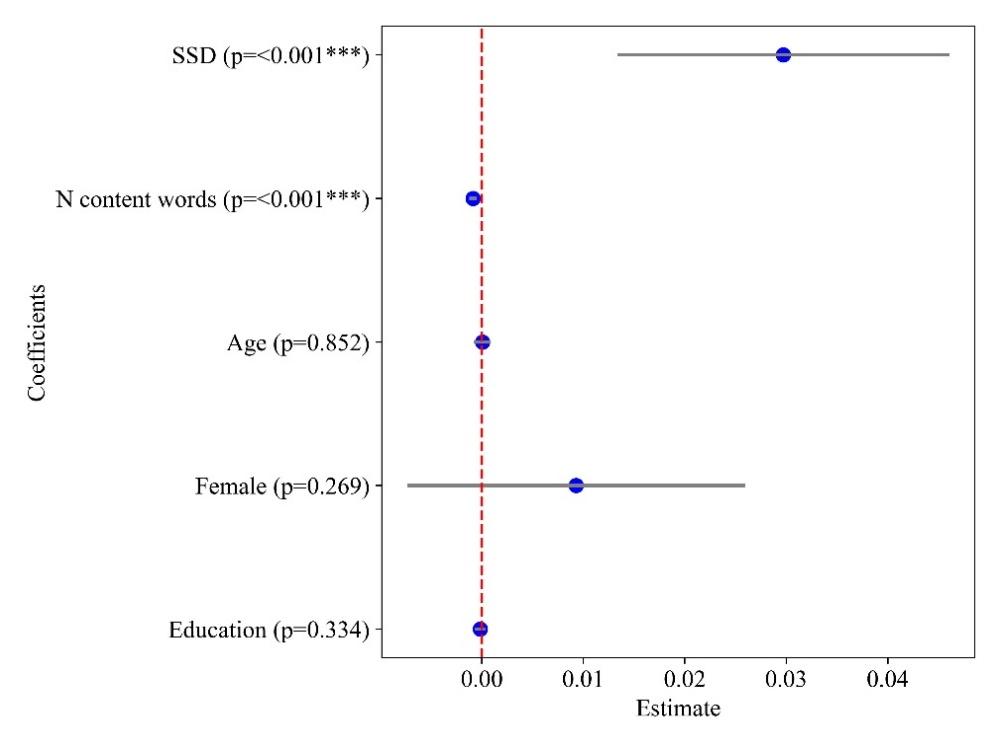 |
| --- |
| B  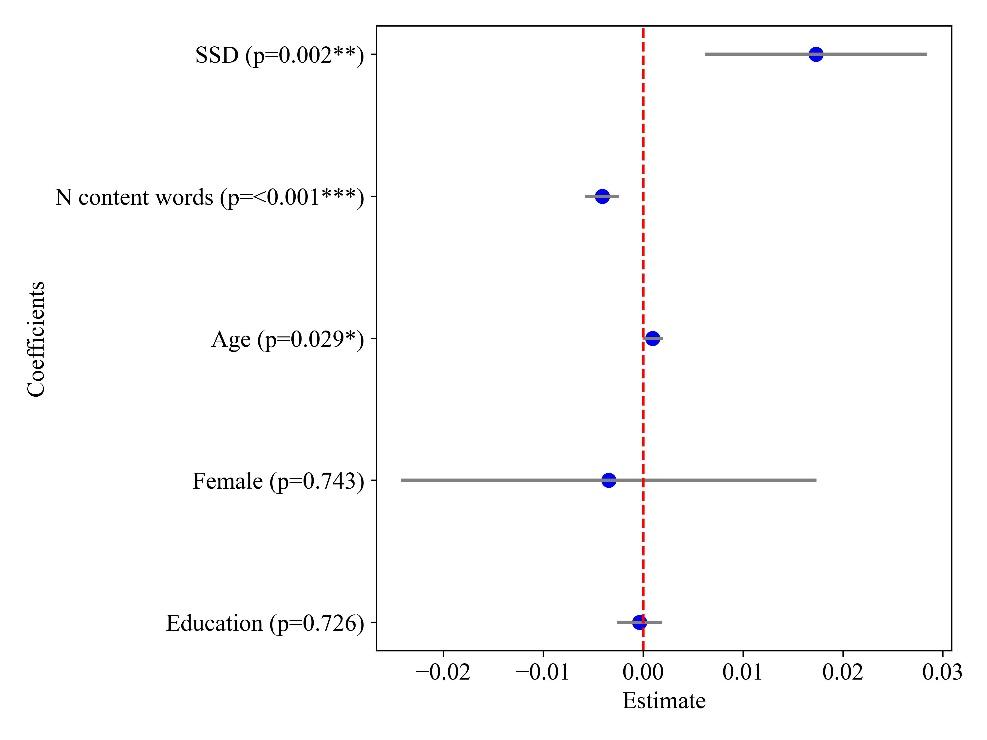 |
| C  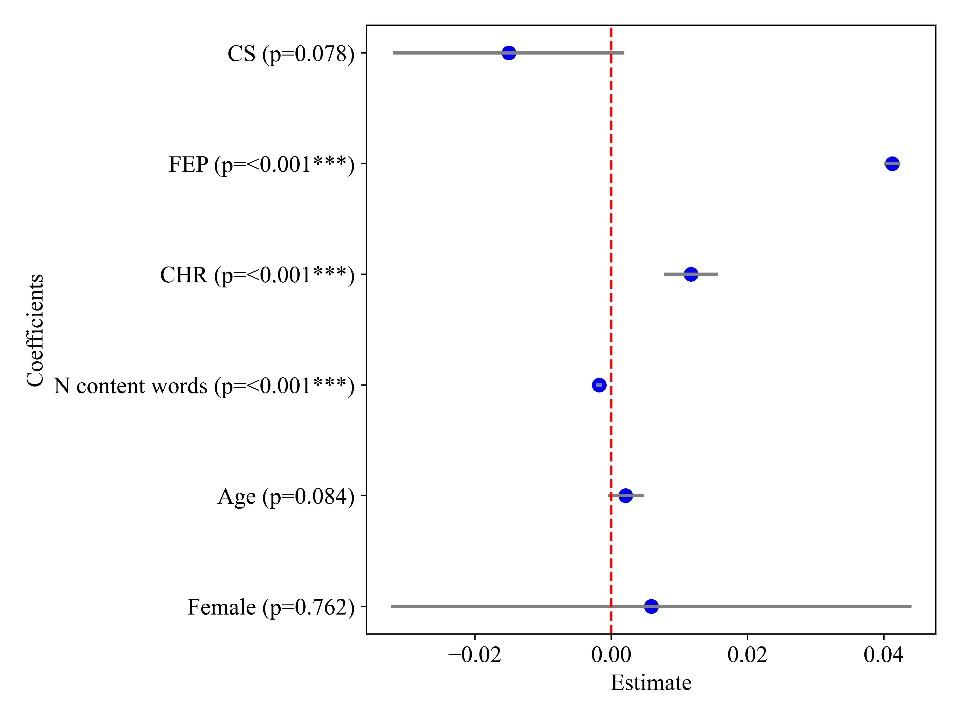 |

**Figure S3**: Estimates of coefficients for each language and regression model of ${ExVar}_{2}$ using all content words in fastText. (A) German, (B) Spanish, (C) English. SSD: Schizophrenia Spectrum Disorder. CS: Chronic schizophrenia. CHR: Clinical High Risk. FEP: First episode psychosis (untreated).

**Sensitivity analysis**

We conducted a sensitivity analysis of intrinsic dimensionality (*ID*) by varying the number of neighbors *k* in the *k*-nearest neighbors procedure described in the Methods section. As an initial examination, we estimated the *ID* using a range of *k* values across all groups and language samples. Figure S4 illustrates how *ID* changes with increasing *k*, showing the mean values and a shaded band representing one standard deviation around the mean. Visual inspection suggests that the default value of *k*=5 may not be optimal, as larger *k* values tend to yield more stable estimates. However, excessively large *k* values could eventually reduce the sample size discarding speech samples with less word counting, especially when in the unique content words using fastText model.


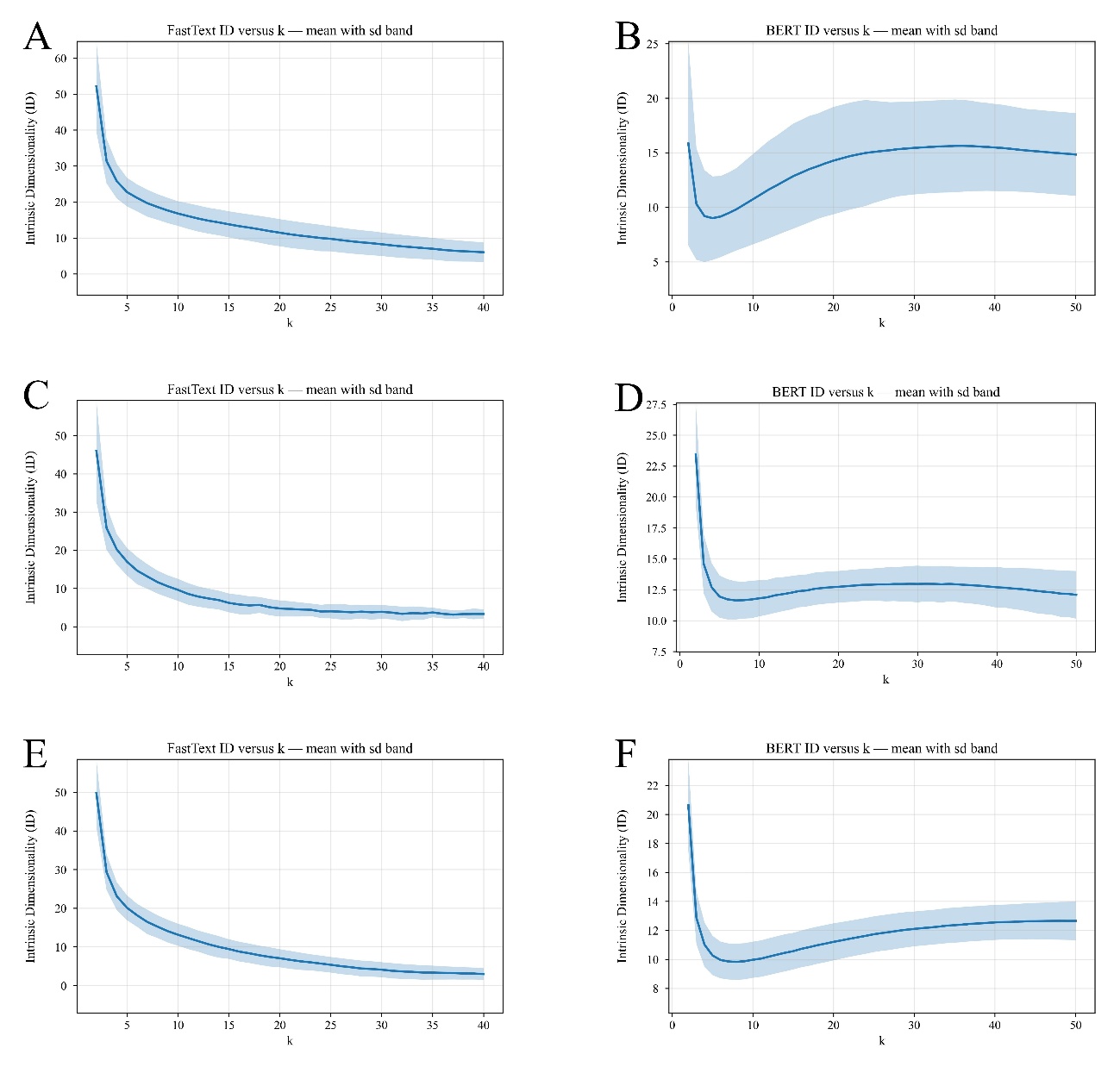


**Figure S4**: Mean and standard deviation (s.d.) of *ID* estimates as a function of the number of neighbors *k*. Panels A and B: German sample; Panels C and D: Spanish sample; Panels E and F: English sample. Left panels (A, C, E) using fastText and right panels (B, D, F) using BERT.

Later, we repeated the regression analysis while varying the number of neighbors *k* in the *ID* estimation to assess the robustness of group effects and their corresponding *p*-values. The results demonstrated a consistent pattern across *k* values in BERT (results in fastText for Spanish sample is not so clear), confirming the stability of the main results. Figure S4 shows the estimated $\beta$ coefficients and associated *p*-values for each model, indicating that the effects remained stable across different *k* ranges (10-25 for fastText and 20-40 for BERT). Red stars denote significant *p*-values below 0.01.


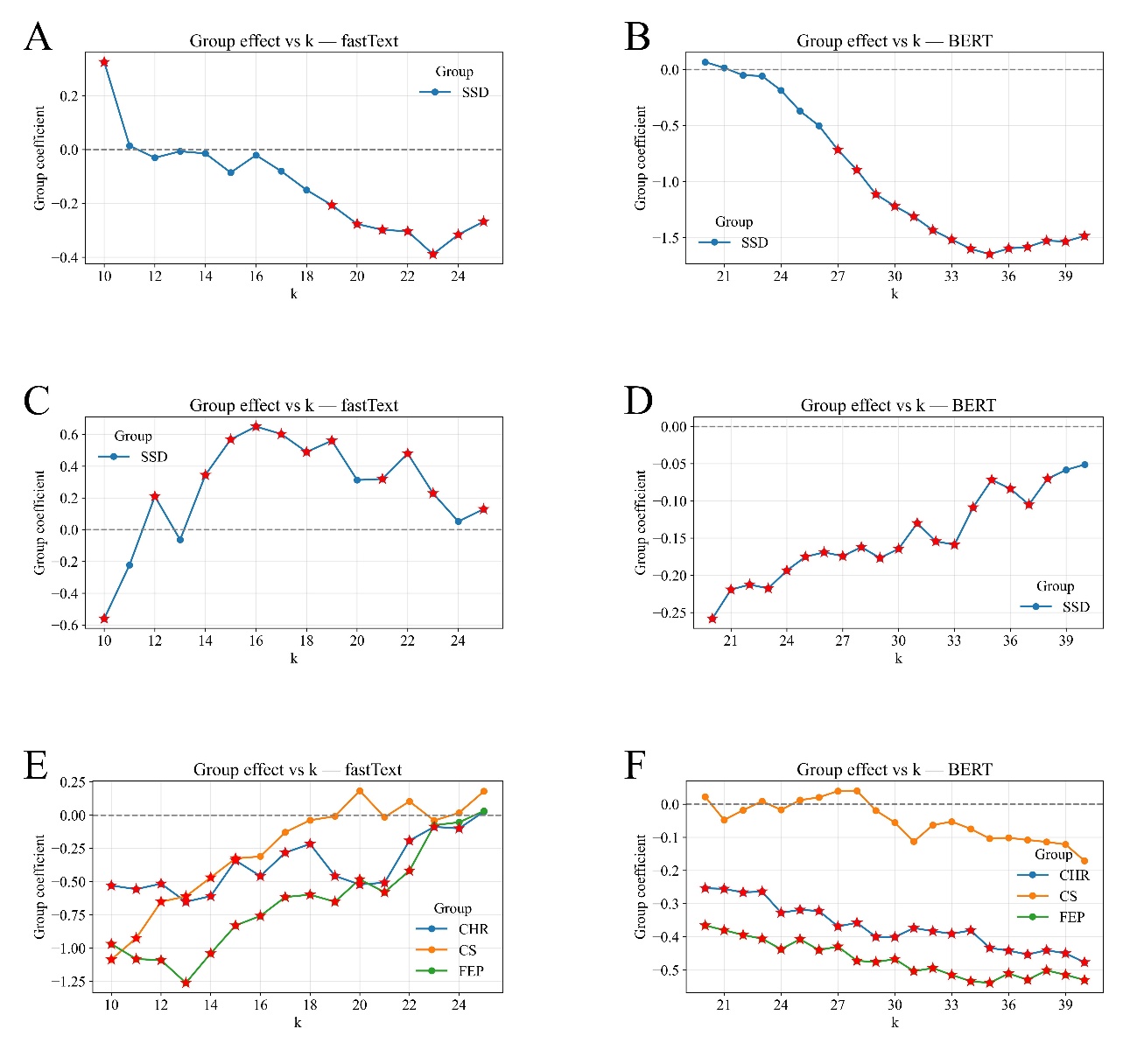


**Figure S5**: Estimated group coefficients as a function of the number of neighbors *k*. Panels A and B: German sample; Panels C and D: Spanish sample; Panels E and F: English sample. Left panels (A, C, E) using fastText and right panels (B, D, F) using BERT. Red stars indicate values of *k* for which the group effect was significant (*p* < 0.01), illustrating the consistency of effects across different neighborhood sizes.
